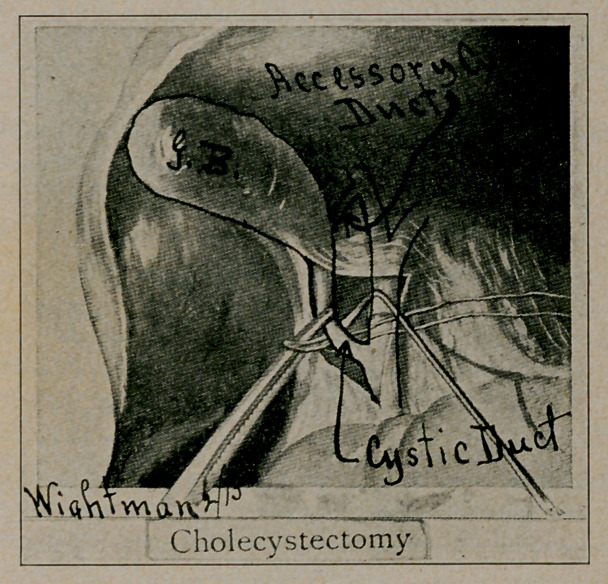# Accessory Cystic Duct

**Published:** 1915-06

**Authors:** 


**Accessory Cystic Duct.** H. W. Wightman, Omaha; Western Med. Review, April, 1915. Report of a case.

Man aged 50 years, presenting symptoms of chronic gall bladder disease. Operation February, 1915.

Gall bladder reduced in size, walls considerably thickened and adherent to transverse colon, lymphatic glands along the hepatic ducts distinctly enlarged. The picture presented all the characteristics of a chronic gall bladder infection.

Cholecystectomy was decided upon, the cystic duct and

Cholecystectomy artery were liberated and ligated conjointly, clamp applied to the gall bladder end and to the cystic duct and artery severed between ligatur and clamp.

While peeling the gall bladder out of its peritoneal bed, the accessory cystic duct was encountered entering the posterior wall at about the lower third of the body of the gall bladder. (See accompanying cut.)

After the removal of the gall bladder, bile could be distinctly seen escaping from the cut end of the accessory duct, which was then pulled out slightly and ligated. Cigarette drains placed in contact with the iodized stumps and wound closed.

Patient made an uneventful recovery and resumed his usual duties sixteen days after the operation.

## Figures and Tables

**Figure f1:**